# Barriers and facilitators for adherence to antiretroviral therapy, and strategies to address the barriers in key populations, Mumbai–A qualitative study

**DOI:** 10.1371/journal.pone.0305390

**Published:** 2024-07-11

**Authors:** Shrikala Acharya, Mugundu Ramien Parthasarathy, Vijaykumar Karanjkar, Sachendra Katkar, Maninder Singh Setia

**Affiliations:** 1 Mumbai Districts AIDS Control Society, Mumbai, India; 2 Seth GS Medical College and KEM Hospital, Mumbai, India; 3 Project Accelerate, Johns Hopkins University School of Medicine, Mumbai, India; 4 MGM Institute of Health Sciences, Navi Mumbai, India; United States Agency for International Development (USAID), NIGERIA

## Abstract

**Background:**

Even though quantitative studies have described barriers to anti-retroviral therapy (ART), a more exploratory approach will provide in-depth information on these issues, and potential suggestions to address these issues at individual as well as structural level. We designed this qualitative study to examine the barriers and facilitators for antiretroviral therapy adherence in key population (KP) in Mumbai, India. We also wanted to understand the strategies adopted by these groups and get suggestions to improve adherence to ART.

**Methods:**

This is a qualitative analysis of seven focus group discussions (FGDs) conducted with four KP subgroups in Mumbai. We conducted two FGDs each with female sex workers (FSW), men who have sex with men (MSM), male-to-female transgendered people/*Hijras* (TGH) each, and one FGD with people who inject drugs (IDU). We transcribed the audio-recorded electronic records of these FGDs. We also added the notes of the observers on the group dynamics to the transcribed data. We used the Framework Approach to analyse these data.

**Results:**

Some experiences–such as side effects to ART medicines–were common across groups. However, incarceration as a reason for stopping ART was reported by FSWs but not by other KPs. Friends and family (including *Guru*) are important support systems for HIV infected individuals and adherence to ART. Stigma and discrimination by community members and general community prevent regular access of ART centres and other health care facilities. Additional factors which led to missed doses were mental health issues, alcohol use, and misplacing the ART tablets during police raids or during robbery attempts at the cruising sites. Since a common source of discrimination among peers and the community was the presence of ‘Green book’ (or their treatment book); the key population wanted the AIDS program to change it to digital cards so that labelling one as ‘HIV positive’ for being seen with the book can be avoided.

**Conclusions:**

The qualitative study helped us explore the barriers to ART among key population and the community provided specific suggestions to address them. In addition to Key Population centric enhanced adherence counselling, some administrative guidelines and procedures may need to be altered to improve adherence to ART in these populations.

## Introduction

The National AIDS Control Organisation (NACO) of India was started in 1992 to implement HIV programmes in the country. Its initial phase included activities such as awareness programmes, blood safety, condom promotion, management of sexually transmitted infections, and surveillance programmes [1]. In the second phase of National AIDS Control Programme (NACP) (1999–2006)—targeted interventions (TIs) for key populations such as female sex workers (FSW), men who have sex with men (MSM), and people who inject drugs (PWIDs) were initiated and have been continued till date [1]. In 2004, India started free anti-retroviral therapy (ART) programme under its national programme at select government centres. Though initially it was available to those with a CD 4 count of <200, it changed over time, and in 2017, India adopted the test and treat policy [2, 3]. Along with an increased roll out of ART services, NACO also supported building of institutional capacities to handle advanced HIV disease, opportunistic infections, and laboratory infrastructure across the country. With an increased use of ART in people living with HIV/AIDS (PLHAs), an emerging issue of concern is adherence to ART. NACO advocates an optimal adherence of >95% for maintenance of low viral loads and high CD4 counts [4].

Poor adherence is associated with inadequate control of viral loads, drug resistance, and progression to AIDS in PLHAs [5–7]. Good adherence, however, is associated with overall good public health outcomes and cost-effectiveness of the programme [8]. Some factors which affect ART adherence are: knowledge about ART, side effects, forgetting doses, or unavailability of ART in the centres [9, 10]. Other factors such as stigma and discrimination, family support, self-efficacy, and desire to live longer also have a major effect on ART adherence [7, 11]. Even though ARTs are provided free of cost at government-controlled ART centres, financial difficulties and food insecurity may also be associated with poor adherence in PLHAs [7, 12]. Key population groups (such as FSWs, MSM, PWIDs, and male to female transgendered people/Hijras [TGH]) may be more prone to stigma and discrimination not only due to their professional or sexual behaviour but also due to their HIV status. This discrimination and anticipated stigma may be at an individual level or at a structural (in the health care or social system) level [13–16]. In addition, many of these individuals may stay away from their family members or may have inadequate social support. Even if they stay with their family members, they may not disclose their HIV status or may not be ‘out’ to their family. It has been reported that disclosure to family members improves ART adherence through social support and self-efficacy [17]. They may also have mental health issues, experience violence, and report substance use (alcohol use and drug use); these factors may also be associated with poor adherence [18–22]. Thus, many of these of the issues related to adherence in key population may be inter-related. All these factors must be included while designing programmes for improvement of adherence in these groups.

Enhanced adherence counselling (EAC), which includes assessment of baseline adherence and needs, provide adherence counselling, educational sessions, and follow-up sessions has been recommended for improvement of adherence in PLHAs on ART [23, 24]. Indeed, studies have shown that EAC is helpful in improving adherence, optimal switching to new ART regimens, and viral load suppression in both key population and general population [25, 26]. Authors have also recommended that interventions in India should focus on mental health issues to improve adherence [27]. Furthermore, a systematic review of EAC interventions found that individual-tailored programmes are more effective in addressing adherence issues [28]. Another recommendation from this review was that interventions should also address structural and institutional issues to adherence [28]. Even though quantitative studies describe the main barriers and facilitators to ART adherence [29, 30], a more exploratory approach will provide in-depth information on these issues, and potential suggestions to address these issues at individual as well as structural level. Furthermore, involvement of the community will improve acceptance of the interventions by key population.

With this background, we designed this qualitative study to examine the barriers and facilitators for ART adherence in key populations (FSWs, MSM, TGH, and PWIDs) in Mumbai, India. We also wanted to understand the strategies adopted by these groups and suggestions to improve adherence to ART in them. The findings of such a qualitative study can be used to develop strategies and interventions aimed at improvement of ART adherence in the key population.

## Methods

The present report is a qualitative analysis of seven focus group discussions (FGDs) conducted with four types of key populations in Mumbai.

### Study site and population

Mumbai Districts AIDS Control Society (MDACS), the nodal organization for implementation of HIV control and treatment activities as per NACO guidelines, operates 26 (current 23) targeted intervention (TI) projects for the key populations through community-based organizations (CBOs)/non-governmental organizations (NGOs) across the city of Mumbai and its suburbs. These interventions focus on HIV prevention activities through outreach services for behaviour change, condom promotion, regular health check, and STI/HIV screening along with community mobilization efforts. The participants for the FDGs were identified with the help of programme managers of these TIs. The participants were purposively selected from the TIs. The research team has also been working with these TIs for the past 10 years and they are familiar with the population. We tried to include some KPs who have started ART recently, some who have been on ART for a long time, some who have maintained >95% adherence, and some who have had a lower adherence. The composition of the FGDs were as follows: 1) TGH—two FGDs with 22 total participants. 2) MSM—two FGDs with 16 total participants; 3) FSWs- Two FGDs with 14 total participants; and 4) PWIDs–one FGD with seven total participants. All the participants who were invited to participate came to the respective FGDs.

### Conducting the FGDs

The FGD was conducted in a safe space in the centre. Before initiating the FGD, we explained the purpose of the study in Hindi and/or Marathi (common languages spoken in the area). The procedures (including audio recording of the FGD) were explained and a written informed consent (in the language they are able to understand) was taken before starting the FGD.

The FGD was conducted by a facilitator (MSS—MD and PhD). He is trained in qualitative research methods and was a part of the research team. The FGDs were conducted in Hindi and Marathi (as discussed earlier these are the common languages spoken in the area). The researcher spoke both these languages and did not require any translation. The duration of each FGD was about 90–120 minutes. They were also provided refreshments in these FGDs. A guide was prepared, and the topics were selected after discussion with the programme managers at the TIs, counsellors at the ART centres, and from a previous quantitative study conducted by us [29]. The topics were: 1) perception and knowledge about ART including side effects; 2) regular time of ART and adjusting to daily activities; 3) issues of disclosure, stigma, and discrimination in community and work place settings; 4) mental health concerns; 5) substance use; 6) any other factors related to poor adherence; 7) facilitators of adherence; and 8) the way they address some of these adherence related issues; and 9) suggestions for addressing some these barriers. These FGDs were audio-recorded.

All the participants were asked to respond to each of these themes, barriers for adherence, strategies that are adopted by the KPs or that are most likely to work to address these issues. The FGD method was useful to understand the issues of communities or group dynamics within a community. Since it was a group, we were able to get different or contrasting views on the same topic at the same time. However, we did face some difficulties while conducting the FGD. For example, sometimes there were heated arguments during the FGD. Some members of the group always contradicted others, while others were quiet throughout the FGD. Whenever such a situation arose, the facilitator stepped in, was polite yet firm while dealing with these, and made the group come back to the main topic. The facilitator also encouraged those individuals who were quiet to comment on the topics. Apart from the facilitator, we had a note-taker in the FGD. They took notes of the conversation during the FGDs and recorded any agreements or disagreements within the group for suggestions and strategies. These notes were also used later during the analyses phase.

### Data analysis

We transcribed the audio-recorded electronic records of these FGDs. We also added the notes of the observers on the group dynamics to the transcribed data. We used the Framework Approach to analyse the focus groups [31]. This method of analysis uses a deductive approach in contrast to the Grounded Theory, which uses an inductive approach. The analyses started deductively according to the aims and objectives of the study, and proceeded systematically to identify the themes and sub-themes in the data collected. The researcher (MSS) was primarily responsible for coding and analysing the data; these themes and sub-themes were also shared with the other researcher (SA).

We read the transcript thoroughly and familiarised ourselves with the contents of the transcribed text. We then proceeded with identifying the thematic framework. We had identified some a priori themes; these were examined in detail in the data. We also defined additional themes according to the issues discussed by the participants. At the end of this process, we identified concepts in the major themes, and sub-themes. This was followed by indexing of the data. We applied the index systematically to the transcribed textual data, and annotated the text with numerical codes. This was followed by charting of the data. We rearranged the indexed data according to the thematic framework. Related items were placed under appropriate sub-themes. Finally, we used these charts for mapping and interpretation, by defining the concepts in the themes, range, and nature of issues covered in various themes and sub-themes, and identify associations between different themes to create typologies and explanations. Some of the main themes were (barriers–subtheme: time, type of medication, family related issues etc.) Thus, the above methods helped us in the content and thematic analysis of the focus groups to identify relevant issues and strategies in the major themes. The analyses were done separately for each high-risk group. However, we compared the similarities and differences across these groups. We used MAXQDA 2020 (VERBI GmBH) for analysis.

The study was approved by the Institutional Ethics Committee of the MDACS (Reference No: 001/2019). All the potential study participants were explained the study protocol in detail, and they signed a written informed consent. As indicated earlier, they were identified though targeted intervention programmes, and they knew the organisation and the personnel in the organisation who managed these intervention programmes.

## Results

The mean age (SD) of study participants was as follows: TGH– 34.4 (8.0) years; MSM– 37.6 (10.5); FSWs– 36.9 (4.7) years; and PWID– 37.7 (10.6) years. We have presented results for each group separately.

### I. Female sex workers

#### I. a. Initiation and continuation of ART

Most FSWs were informed about benefits and side effects of ART at the start of treatment. However, individuals still complained about side effects, particularly, in the first one month after initiation of the ART. The common side effects were–headache, giddiness, drowsiness, and vomiting. Though, one individual stopped the medicines due to vomiting, in general most of them continued to take them after receiving assurances from doctors and counsellors that most of the side effects will subside after some time. Only one FSW reported that she did not receive any information. Another FSW informed us that she was concerned about the size of the tablet and wondered if smaller substitutes were available for the same. FSWs themselves encouraged others to start the treatment, and they informed other positive women about ART including their own experiences.

*“I told my example to two positive women in my community and help them to start ART*. *Now their health is good*. *They are going to work”*

- FGD Participant

*“Doctor told me to take complete rest. He gave me information about diet. He told me that you will have side effect for few days, but do not leave medicine”*

- FGD Participant

*“I did not get any information from hospital. When I had side effects, I asked doctor. He said, it is because of tablet, I will change it”*

**- FGD Participant**

Support from friends and family members was an important factor in initiation and appropriate intake of medicines. In general, most FSWs had support from their family members–either husbands/partners or children. In fact, disclosure to their family members had helped them to take medicines on time.

“*When my family was not aware of my HIV status that time*, *I used to skip tablet*. *Now*, *everyone knows about it"*

- FGD Participant

*“I had to hide my tablet from my family members. Now I have told them. I eat tablet in front of them*.

**- FGD Participant**

Another advantage of initiation of ARTs was maintenance of anonymity. One participant found that prior to ART initiation, she frequently visited the hospital for health problems. Her neighbours were inquisitive about her disease due to frequent hospital visits. After starting ART, she does not have these health problems; thus, the neighbours have stopped enquiring about her health.

#### I. b. Fear about ART, missing doses, and strategies used to ensure adherence

One major concern was that others will know about their HIV status because of ART drugs. Thus, they store their medicines in purses or vessels, or hide them between clothes. In fact, they also hide their ‘Green Book’–the record book used for ART visits and treatment. Indeed, one FSW informed us that she keeps the book with the organization. Another FSW informed us that a health care worker from the hospital came to her home for follow-up in her absence and started enquiring about her whereabouts. The FSW was concerned that her neighbours would come to know about her HIV status. Then, she told them firmly not to come to her home ever again. Some FSWs are scared to go the nearby ART centre for medicines–‘someone may see them at the centre’.

“*One day there was blood check-up in the school*. *I told them I will not send my daughter for blood check-up*. *I will do it in private pathology*. *That day I did not send my daughter to school*. *At the age of thirteen her positive status was detected*”

- FGD Participant

Another reason for missing these medications was ‘police raids’; they are taken to the police station. They are likely to miss ARTs whenever they are incarcerated.

“*Once police arrested me at 7*.*30 pm*. *My ART time is 8 pm*, *I fought with the police and told them that I am HIV patient*, *and I have to go home and take my tablet*. *As soon as they heard of HIV*, *they allowed me to go home*”

**- FGD Participant**

Many of them reported that they take medicine on time, usually one hour after they eat food. They have been counselled to decide on one fixed time and ensure that they take the tablet around that time. However, these FSWs were not aware about the maximum desirable time beyond the designated time for taking these medicines. They suggested the use of alarm clocks as a reminder or anchoring their ART intake to a television series. Most of them had developed their own strategies to ensure adherence to medications. They carried medications whenever they went out (even to their native places), and always kept extra medicines in their purse.

#### I. c. Alcohol/drug use and mental health concerns

One participant reported that FSWs who used alcohol and drugs were less likely to use condoms during sex. Though, some of our participants used tobacco (oral/chewing form), they did not consume alcohol or had stopped after starting ART. Fights with partners, friends, and customers were common; and some participants reported missing medications after these fights. They were aware of the harmful effects of missing drugs. Thus, they wanted provision for counselling due to stress or mental health concerns. Even though the counsellors and doctors at ART centres were helpful, it will be useful to include professional mental health counselling.

#### I. d. Disclosure and sexual behaviour in HIV infected FSWs

Some of these FSWs were not comfortable in disclosing their status to anyone; they feared that others will stop interactions with them. One of them was angry about being infected, and her partners had deserted her due to her status.

*“Why should I tell customer about my HIV infection? My first husband was infected*, *so I got this infection*. *My second partner left me when he came to about my HIV status*.*”*

**- FGD Participant**

However, not all FSWs were concerned about disclosure of their status to others. In fact, one FSW informed us that even though the counsellor had suggested confidentiality, she did not feel the need for it. Another FSW informed us that she does not hesitate to take medicines in front of clients. The HIV infected status was most commonly disclosed to family members including children; they were a strong support system for ART intake and hospital visits.

Many FSWs were aware about re-infection due to unprotected sex, as this was discussed in the counselling sessions. Hence, most of them encouraged the use of condoms with their sexual partners. Some others are willing to have unprotected sex with some sexual partners–particularly if the partner insists or is ready to pay more for sex. Furthermore, some of them used condoms with their regular partners (boyfriends) but did not insist on their use with their customers.

*“Some FSWs allow customers to have sex without condom if they offer more money*. *But some refuse to do without condom even if they get more money”*

**- FGD Participant**

Certain key points about FSWs are presented in Box 1.

**Box 1. Key findings from the focus group discussions conducted with female sex workers (FSWs).**

**KEY FINDINGS FROM THE FSW GROUP DISCUSSIONS**

• Side effects are common especially in the initial month, but FSWs are aware of them; few FSWs stop medications due to side effects

• Size of the tablet is a big concern; it stands out if they have it in front of others

• ‘Green Book’ is a source of anxiety; they try to hide their medicines and the Green Book

• ART is usually taken on time (due to alarm clock or television series); however, the maximum desirable time beyond the designated ART time is not known

• Tobacco use is common (chewable form); however, alcohol was not commonly reported in those on ART

• Mental health counselling is required on a regular basis

• Re-infection prevention needs and regular condom use—particularly with ‘paying partners’—needs to be strengthened in the counselling process

• Strategies to ensure ART during incarceration are important and SOPs should be in place

• Family is a strong source of support and involvement of family members may be considered in improving adherence and medical care

## II. Men who have sex with men

### II. a. Initiation and continuation of ART

Though many MSM were informed about ART and side effects, few were not adequately counselled about the process of ART initiation. One participant reported that he was very motivated to start the ART, and he convinced the counsellor and doctor to start the treatment immediately.

The participants reported that they experienced lot of side effects immediately after starting ART. The common side effects were vomiting, giddiness, bad dreams, and tingling and numbness. Though, some MSM stopped ART due to side effects, it was usually temporary, and they restarted after the side effects subsided. Moreover, some thought that they were healthy till the time they had started the ART; however, once they started ART, their health worsened due to side effects. Thus, they were likely to stop ART as they associated initiation of ART with poor health.

*“Yes*, *there are people who stop medicine because of side effects*. *Because before starting ART they were fine*. *After starting ART situation is bad*, *so*, *they stop*.*”*

**- FGD Participant**

We also found that some MSM do not start ART even after knowing their HIV status; they are reluctant to accept their status and start medicines. Thus, it was suggested that doctors and counsellors should stress on the importance of medicines and convince all MSM to start ART. They should counsel about ART in simple language, so that MSM are able to comprehend it completely.

*“When they start ART*, *they give information*. *They are very fast when they talk*, *if we don’t understand and ask again*, *they said you should listen carefully*.*”*

**- FGD Participant**

Support from friends and family was mixed. While some MSM found the family to be supportive, others found that they did not care much and even discriminated against them. Some family members take MSM back to villages and start treatment from rural hospitals. Friends, however, are a bigger source of support. NGOs and TIs are very important support systems for HIV positive MSM. NGOs give them information about HIV and ART, and provide safe space. Furthermore, counsellors at the NGO are more considerate and some of the MSM even receive calls for ART reminders. Thus, in general, they had a positive view of NGOs and TIs. An important suggestion was to develop mechanisms for family counselling, since some participants who are married and have supportive family are more likely to have positive health outcomes.

*“I would like to suggest that MSM family also requires counseling*. *Family should not demotivate KP”*

- FGD Participant

*“When ART counselor talks to me, she asked my sister-in-law to sit out. Counselor could have talked to her as well”*

**- FGD Participant**

### II. b. Fear about ART, missing doses, and strategies used to ensure adherence

MSM were concerned that they may be identified as HIV infected due to these ART medicines. Hence, they use multiple strategies to hide them. They remove the sticker on the bottle. They keep some additional tablets in the same bottle so that others are not able to recognise the medicines, or put all these medicines in a multi-vitamin bottle.

*“Some people change tablet box and then take it*. *If anyone asks*, *then they lie*. *But he cannot openly say that it is for HIV”*

**- FGD Participant**

A major area of concern is the ‘green book’. This is the record book for all HIV infected individuals registered at the ART centre. They were concerned that others will come to know about their HIV status if they see them with this green book. Indeed, participants informed us they have faced discrimination at workplace due to this book. Thus, many prefer to keep these books with the TI or NGO. They are also scared to go ART centres for the fear of being recognised by other MSM in addition to missing a day’s work.

*“I have to take leave from office if I have to go for medicine*. *I have to hide my book in my bag*. *I lost my two jobs because they found green book in my bag”*

**- FGD Participant**

Most participants were quite regular about medications. They were not aware about the maximum desirable time to take medicine after missing the dose. They carried their medicines along with them; however, one participant informed us that he misses ART whenever he is out for sex work. Alarms were commonly used to remember intake of medicines. Friends, family members, and NGO representatives also reminded MSM about medications.

*“When I go for sex work*, *I am not able to take medicine on time*. *My ART time is 10pm*, *when I take medicine*, *I feel sleepy*. *Then I won’t be able to do sex work*. *I told this problem to doctor”*

**- FGD Participant**

### II. c. Alcohol/drug use, mental health concerns, and other issues

Alcohol and drug use were common in MSM in TIs. However, they are not counselled about alcohol or drugs, and concomitant use of ART. Often, MSM change their time of ART after consuming alcohol. Our participants informed us that they usually consume alcohol whenever they go for sex work and due to this they were more likely to miss ART. Drug use is also common in MSM, particularly among the more affluent ones. However, there is little counselling about alcohol and drug use in these KPs.

*“I can do sex work only after drinking alcohol*, *otherwise it won’t be possible”*

- FGD Participant

*“There are drugs addicts they are taking ART and drugs also. Our doctors need to understand these issues. It is not east to stop taking drugs. One can stop alcohol but impossible to stop drugs. These button drugs they put with any cold drink. They take it and stand near bus stop with short clothes. Thinner, Whitener, Corus are also types of drugs”*

**- FGD Participant**

Fights with family members and partners are other important reasons for missing ART doses. Some are also fatalistic about their condition. Thus, there is a concern about mental health, and there is a need to improve this aspect of counselling. MSM also face discrimination because of their HIV status; this also adversely affects their mental health.

Apart from discrimination by family members and community, the hospital procedures in ART initiation and continuation are also responsible for discrimination experienced by them. As discussed earlier, ‘green book’ is a big source of stress and MSM take all precautions to hide it from everyone in the hospitals. Sometimes, MSM are asked to paste their photographs on the book and they are not comfortable with it. It was also highlighted that some MSM do not want test for HIV even if they have had high risk behaviour. They fear discrimination after being detected HIV infected.

*“Once a child from family opened my bag and took my book out*. *I immediately put it inside*. *I was so scared that time”*

- FGD Participant

*“If we carry book with us then people come to know that we are on ART”*

**- FGD Participant**

Food intake was another important concern and the participants told us that food supplements were an important intervention for HIV infected MSM. They used to receive nutrition supplements from an NGO. However, since it was a part of the funded programme these supplements stopped after completion of the project. They suggested that these supplements should be restarted and should be done at the ART centre or in NGOs/TIs. Few MSM are not comfortable with home visits of NGO outreach staff due to stigma associated with HIV infection.

### II. d. Disclosure and sexual behaviour in HIV infected MSM

Most MSM do not disclose their status to family members or other friends; they may be comfortable disclosing it to other HIV positive individuals. However, they are scared to visit ART centres for the fear of being noticed by other members of the community. Even if they disclose, they do it after a couple of years of detection.

Most of them were aware of re-infection and used condoms with sexual partners. Though, some are offered more money for sex without condoms, most MSM insisted on condom use. Many participants reported a reduction in sexual desires and activity, and wondered if this was due to HIV infection or ART. It was also reported that some MSM may inject drugs and share needles; thus, they may be prone to re-infection due to injecting behaviour rather than sexual behaviour.

Certain key points about MSM are presented in Box 2.

**Box 2. Key findings from focus group discussions conducted with men who have sex with men (MSM).**

**KEY FINDINGS FROM THE MSM GROUP DISCUSSIONS**

• Side effects are common and many MSM may stop medicines due to these side effects. Reduction in sexual desire was also reported in those on ART; many of them attributed it to either the infection or the treatment

• ‘Green Book’ is a source of anxiety and discrimination; they are not comfortable carrying it to the hospital or keeping it at home

• Family support may not be available to all KPs; however, they suggested to include family members in counselling sessions to sensitise them to HIV and ART.

• NGOs and TIs are a stronger source of support to these KPs; however, some may not be comfortable if they make home-visits

• Strategies to include alcohol-related counselling and risk-reduction due to injecting drug use should be an important component of the MSM TIs

• Stress and mental health issues due to infection, stigma, and discrimination by friends and family members are common in the community

• Though safe sex is practiced by HIV infected MSM, unsafe behaviour seems to be incentivised, particularly among sex workers in the form of additional money

• Fear of accidental disclosure to other KPs at ART centres may hamper regular HIV care access

## III. Male to female transgendered people/hijras

### III. a. Initiation and continuation of ART

Most TGH report side effects after initiation of ART. This is usually in the first month of starting the medications. Some of the reported side effects are giddiness, tiredness, irritability, sleepiness, and skin rashes. Many TGH stop medicines due to these side effects and some are hesitant to take/restart the medicines due to the size of the tablet. Furthermore, some TGH also think that medicines from private clinics are better compared with those available in government hospital

Another reason for stopping ART is perceived or actual effects of these medicines on physical appearance. The participants informed us if individuals have any skin changes or changes in the physical appearance, it is attributed to initiation with ART and is discussed within the community. Many TGH may stop ART medicines due to these discussions. It has also been observed that if they feel better after initiation of ART, then they stop medications. Thus, it was repeatedly suggested that all TGH should receive detailed counselling every six months.

*“TGH should be beautiful*. *After starting ART TGH observes physical changes*, *like change in skin*, *big stomach and some more problems*. *When they lose their beauty*, *they talk to each other that this happened because of ART”*

- FGD Participant

*"Some TGH take ART for six months; once they feel healthy, they leave medicine”*

**- FGD Participant**

Supportive Guru (who is the head of TGH group/adopted family), family members, partners, and friends are useful to ensure adherence to the ART. Thus, it may be worthwhile to include them in counselling sessions if the TGH agree.

### III. b. Fear about ART, missing doses, and strategies used to ensure adherence

Some TGH were concerned about taking medicines in front of others, particularly if they think that they will discriminate against them due to their HIV positive status. Hence, they try to hide their medicines in other boxes or remove labels of the ART box. If someone asks them about the medicines, they just reply that it is for some other health condition–such a blood pressure, diabetes, or indigestion. However, they are concerned that their status may be revealed to other community members and wondered if the pills can be provided in different boxes such that other community members are not able to identify them as HIV medicines.

*“If the packing is different then it will be easy for KP to take medicine”*

**- FGD Participant**

Most of them take their medicines on time. They use alarms as reminders or their friends/family members remind them. In fact, one participant informed us that she lives with three other HIV positive individuals. They all take their medicines together. Thus, HIV infected TGH on ART can be role models for those who have been newly detected. Some participants suggested that HIV infected TGH can be made counsellors at the ART centres. Earlier, many TGH were not ready to disclose their HIV status; hence, it was difficult to form support groups for PLHIV. But now, more TGH are comfortable discussing their HIV status and are willing to attend HIV support groups. Though messages and phone reminders are important to ensure adherence, everyone may not be literate. Thus, it was suggested that pictorial messages may be more useful compared with text messages.

*"HIV Positive TGH should give their example to other TGH*. *Tell them that when you started ART you also have the same problem for few days*. *After that everything is ok”*

- FGD Participant

"I am not educated. I cannot read mobile message”

***- FGD Participant***

Another important reason for not being able to take medicines on time and ensure good adherence was misplacing the ART bottle. Sometimes, *‘cheats’* (thieves who act as clients of TGH) and bullies may attack and rob TGH at the cruising sites. Since many carry TGH their ART boxes with them, their boxes are also stolen. If they ask for a replacement ART box at the centre, they are required to produce an official police report. If they are not able to produce the report, then the ART bottle is not replaced. If TGH do not take medications for few days and feel fine, they are less likely to continue medications in future. Thus, non-replacement of lost medications may result in poor adherence in future.

*"If some one lost the tablet box*, *then he will not get medicine for next one month*. *Then he thinks that nothing happen to him though he has not taken ART for one month*. *This incident pushes the habit of skipping tablet"*

***- FGD Participant***

### III. c. Alcohol/drug use, mental health concerns, and other issues

Alcohol use is common among TGH, particularly in those who are in sex work. Many TGH forget to take medications after alcohol consumption. However, there is limited counselling on the role of alcohol in ART adherence, or the interaction between ART and alcohol. Drug use was, in general, not common among TGH.

*“Many TGH don’t take ART after taking alcohol*. *We didn’t get information about it”*

***- FGD Participant***

Mental health is another important issue. Sometimes, they fight with friends or partners, and often feel low. They may consume alcohol after these episodes. However, there is limited counselling for mental health and anger management issues; these aspects are important and should be included in counselling sessions. Sometimes, they are also discriminated by other TGH due to their HIV positive status. Thus, they may not always get support from other community members. Many TGH feel discriminated at the ART centres. They feel that counsellors and doctors are not polite with them and there spend a lot of time at these centres.

### III. d. Disclosure and sexual behaviour in HIV infected TGH

Disclosure is not easy due to discrimination by partners and other community members. Hence, many TGH do not disclose their HIV status even to their regular partners due to the fear of being abandoned by them.

It was reported local *gundas (strongmen)* may have forced sex with TGH without condoms. Even if the TGH try to tell them about the risk of unprotected sex, these local bullies do not listen to them. Some TGH may have unprotected sex due to additional monetary compensation. However, many are not aware about re-infection or other sexually transmitted infections.

Certain key points about TGH are presented in Box 3.

**Box 3. Key findings from focus group discussions conducted with male to female transgendered people/*Hijras* (TGH).**

**KEY FINDINGS FROM THE TGH GROUP DISCUSSIONS**

• Side effects are common and many TGH may stop ART due to these side effects. One major concern was loss of physical beauty due to skin changes and body shape after initiation of ART

• They need to be counselled about ART and side effects every six months

• Supportive Gurus are useful in initiation of the ART and ensuring adherence; they may be included in the strategies for improving adherence

• TGH living with HIV/AIDS may be included for group counselling; they may share their experiences

• Guidelines/Procedures for lost ART should be developed and clearly displayed in all ART centres

• Discrimination by the community members is an important concern; programmes to sensitise the community may be included as a part of TI interventions

• Alcohol use is common; training modules for alcohol counselling in HIV infected individuals should be developed and this should be regularly done at TIs or at the ART centre

• Strategies for disclosure and risk-reduction counselling in HIV infected individuals should be included in the TGH programme

## IV. People who inject drugs

We conduced only one FGD with PWIDs. We have presented all the findings this section.

Most PWIDs were counselled adequately before initiating ART. However, they were not informed about certain side effects–such a loose motions. Some of them felt unwell after stopping ART and they restart them. When PWIDs visit their native place, they stop ART for a longer period of time. They are concerned about disclosure of their HIV status to others in the village; this may result in discrimination by other members of the community.

Support from NGOs and family members help them continue ART. Since many of them stay away from their families and are homeless, support from family is not available to all PWIDs. Hence, NGO support will be more useful for this population.

*“When we stay with the famil it is a different situation—you get food to eat*. *But we stay on the footpath*. *We don’t get food to eat*. *We cannot be hygienic”*

***- FGD Participant***

There is a constant fear of double discrimination—due to the use of injecting drugs and being HIV positive. Others also come to know about the medicines due to size and colour of tablets. Thus, there is a lot of stress to hide the medicines. Furthermore, since some of them are homeless and may stay on streets, they may misplace the ART bottle. Thus, there should be some mechanism to involve the TI NGOs in ART adherence strategies.

Most of them have been informed about the importance of taking medicines on time. They usually take multiple drugs and alcohol, and forget about ART medicines. Thus, there is a need to integrate this aspect of counselling in ART centres to ensure adeqaute adherence.

*“Now if we get alcohol*, *we will look for ganja*, *when we get ganja*, *we will look for some other drug*. *And the desire and search go on till the time we are absolutely high*. *That time we forget to have ART*. *Next day I remember that I missed my ART”*

***- FGD Participant***

Those living alone miss their family members and they are likely to forget ART. Thus, they require regular mental health and stress counselling. Even though, family support is important, most of them have not disclosed it to their family members. However, some of them think that family members know about their HIV status due to their health or medicines. Many PWID reported that it was important to disclose their status to their friends and family members. Thus, disclosure strategies should be included in the counselling module.

"*My family doesn’t know about it*. *I don’t know if they got the medicine and read the name of the medicine then they might have known*. *But I am unaware of this"*

***- FGD Participant***

High risk behaviour in the form of unsafe sex and sharing of needles were not commonly reported. However, some sexual partners want to have sex without condoms.

A major problem they face at hospital is repeated pricks by the nurses/laboratory technician to draw blood for investigations. Thus, it may be prudent to assign senior/technical competent individuals for blood collection in PWIDs.

*“There are 8–10 nurses*, *but they don’t get blood*. *They get tired of finding the nerves (veins) but fail to find it*. *They inject the needle so many times in the body that now it pains”*

***- FGD Participant***

Certain key points from the FGD are presented in Box 4.

**Box 4. Key findings from focus group discussions conducted with people who inject drugs (PWIDs).**

**KEY FINDINGS FROM THE PWID GROUP DISCUSSION**

• Side effects are common and many PWIDs may stop medicine due to these side effects. However, visit to the native village is a common reason for stopping medicines

• There is multi-layered stigma–being HIV positive and being an injection drug user; we need to address this in the counselling sessions

• Some of them are homeless and are likely to miss ART due to it. Thus, we need to develop guidelines for NGOs to engage the community and provide medicines regularly

• Polydrug use is common. Hence, the ART counsellors should be trained in different types of drug use and their effects on ART

• Assign senior/technical competent individuals for blood collection in hospitals/ART centres

## V. Suggestions for improvement

One common suggestion was to provide nutrition–such a protein powder—along with ART at these centres or through NGOs. Many community members were of the opinion that AIDS Society should take an active role in reducing discrimination about HIV in the general population. This can be done through online advertisements, online videos, digitial media, and television commercials. There were a lot of commericals earlier, but they are not screened currently. Most advertisements are shown around World AIDS Day on December 01. However, the community members were of the opinion that these messages should be shown throughout the year. One suggestion was to create an app which will provide information about different aspects of HIV. They wanted a toll free number which could link to the AIDS Society. They wanted to use this number to give feedback about ART services.

Another suggestion was to use digital platforms to maintain ART and clinical record using finger prints, rather than the ‘green book’. This ‘green book’ is a source of stress for the KPs and many thought their status is public knowledge because of this book. The label on the medicine box has words such as AIDS Control. Thus, they are not able to hide their status and maintain confidentiality about their HIV status. Thus, they wanted some other box or a general message on the box, so that anonymity about medicines in maintained. Furthermore, they wanted the medicines for longer period (more than one month) every visit. It may be difficult to follow-up every month as they have to take leave from work.

Though condom messages are shown on digitial platforms and television, these advertisements do not specify the role of condoms for prevention of STIs and HIV. They wanted to include this messages in condom advertisements.

## Discussion

These FGDs provided useful information on strategies to increase uptake of ART, and ensure adherence in the community. Though some experiences–such as side effects–were common across communities, others differed. For instance, incarceration as a reason for stopping ART was reported by FSWs but not other KPs. Friends and family (including *Guru*) are imporant support systems for HIV infected individuals and should be included in management of HIV positive KPs. Stigma and discrimination by other community members and general community prevent regular access of ART centres and health care facilities. The community members suggested that digital records should be maintained in the centres so that they do not have to carry their ‘treatment book (green book)’ which may be a source of discrimination. Furthermore, they also wanted MDACS to take a lead role in addressing stigma and discrimation in the society, and set up helpline specifically for ART related issues.

Stigma around HIV, side effects of medications, and support from peers and family members were important factors associated with adherence in these at-risk groups. Stigma amd discrimination in HIV infected key population may be due to sexual orientation, gender affirmation, behaviour or practices, and HIV status; this may affect their health seeking behaviour and adherence to medications. They may experience stigma and discrimination not only by the society but also by their own peers due to their HIV status. Kim and co-workers found that verbal harrasment, rejection by friends, and no help by the police was associated with a perceived stigma in the health care settings among FSWs [32]. They also found that MSM who faced harrasment or were afraid to walk in public were also more likely to perceive stigma in health care settings. It has also been found that men who have sex with both men or women, or married MSM may have experienced stigma or have mental health issues; thus, tailormade interventions to address stigma and improve HIV adherence in these indiviudals are required [33]. Stigma by peers and community members is also an impediment for health care access among sex workers. A study in Kenya found that many sex workers felt that HIV infected sex workers should not sell sex. Furthermore, most of them agreed that sex workers (both male and female) who have apprehensions about HIV-related stigma were more likely to delay their health seeking behaviours [34].

In general, poor social support for PLHIVs is also associated with poor health care access and low adherence among PLHIV [35–38]. In our study, we did find that PLHIV who had social support were more likely to take medications. In these key populations, social support may be different from PLHIVs who are not a part of these groups. For instance, in MSM it may be the adopted family of other MSM or gay men, in FSWs it may be the brothel owner, and for the TGH, it may be key leader of the TGH household or the *Guru*. Thus, counsellors providing services to key population should be trained to counsel about disclosure of HIV-positive status not only to biological families (in key population who stay with their family members) but also non-biological families (for those who stay in community household settings). Addressing various types of stigma (such as anticipated or experienced stigma) from general community, peers, or health care settings should also be included in these training programmes [39–41]. An important cause of anticipated stigma is the ‘green book’—it is a record book which includes details of HIV management and PLHIV are supposed to carry this book during their visits to the ART centre. They can be easily identified as HIV positive when they are with this book. It was suggested by most participants that hiding the large book is very difficult in health care settings; thus, changing these books to digital cards will reduce this stigma.

Some other factors which led to missed doses were side effects of the ART, alcohol use, and misplacing the ART tablets during police raids or when robbed by ‘*cheats*’ at the cruising sites. Studies have found that occurrence of side effects may result in intentional missing of medications in PLHIV [42, 43]. This non-adherence was more pronounced in individuals who had lower knowledge of ART and its side effects, severity of side effects, and self-efficacy about ART medication [44, 45]. Thus, information about mechanism of action of ARTs, their side effects, and importance of adherence should not be a one time session. Rather, it should be an ongoing exercise, at least once in six months, and protocols for regular updates on ART side effects should be included in training manuals for counsellors. In our study, TGH were particularly worried about the side effects of ART on their appearance–for instance body fat re-distribution, and interactions with hormonal therapy. These aspects should also be included in regular counselling sessions with TGH.

Alcohol and other substance use may also be associated with poor adherence [20, 46, 47]. Non-adherence to ART may be more in PLHIV who believe that it may be harmful to mix ART with alcohol [48, 49]. Alcohol use and substance use have been reported in FSWs, MSM, and TGH in India [50–52]. Thus, there is a need to collect information about substance use and counselled about role of alcohol and other psychoactive substances on adherence. It is also important to note that risk behaviours may be not be compartmentalised and often overlap. Thus, some of these individuals may have high-risk sexual behaviours as well as substance use (either orally or injectable). It may be worthwhile to have integrated targeted interventions (sex workers and substance use); this will ensure that counsellors are trained to address both these risk behaviours in the same TI programme. The National AIDS Control Programme should develop guidelines for integrated targeted interventions–particularly in areas and settings where both these high-risk behaviours are common.

Another important issue which resulted in missing drugs was misplacing them during raids or due to theft. FSWs, MSM, and TGH report violence from clients and pimps, partners, ‘*cheats*’, and police [19, 53–55]. It is likely that KPs may lose their ART during police raids, theft and robberies, or during incarceration. The community members informed us that there were no standard practices to replace these lost medicines in the ART centres. Thus, during this time they may skip ART medications and often forget to collect them on time–since they do not have a count of how many pills they have missed. Though a lot of interventions and advocacy groups have highlighted the importance to reduce violence (including strucural violence) in key population, standard guidelines also need to be developed for replacement of lost ART in these situations. These guidelines should also ensure that people don’t take advantage of such lost ART medicine policies, and access multiple doses or doses for those who are not enrolled at these ART centres.

During these FGDs, we did not explore issues such as mental health, stigma and discrimination, social suport systems, disclosure, or violence in greater detail. Multiple facets of all these topics can be studied in each of these groups within the qualitative theoretical framework. However, the main focus of our study was to understand the barriers and facilitator for adherence to ART in key population. Furthermore, we were also interested to know about the actionable points identified by the community. Our intention was to try to incorporate these actionable points within the existing HIV management and care programmes, and modify the existing TI guidelines appropriately.

In spite of the above mentioned limitation, our study provides useful information on the factors associated with adherence in key population in Mumbai and strategies to address some of these barriers. This study was a part of a bigger study to understand issues related to adherence in these high-risk groups; the quantitative findings are presented elsewhere [29]. The qualiative study helped us explore these barriers in detail. Furthermore, discussion with community members also helped us to identify certain actionable points (Box 5); these can be implemeted at the local level or even in the national guidelines. We found that stigma around HIV, discrimination by the general community and peers, side effects of medications, and support from peers and family members were important factors associated with adherence in these at-risk groups. Additional factors which led to missed doses were mental health issues, alcohol use, and misplacing the ART tablets during police raids or robbery attemptsat the cruising sites. A common source of discrimination among peers was the presence of ‘green book’ (or their treatment book). Hence, the key population wanted the AIDS Control Society to change it to digital cards, so that they are not immediately labelled as ‘HIV positive’ by everyone who sees them with the book. Thus, in addition to enhanced adherence counselling, administrative guidelines and procedures have to be modified and updated to improve adherence to ART in key population who are a part of the targeted interventions.

**Box 5. Key suggestions collected from focus group discussions conducted with key population, Mumbai, India.**

**KEY IMPLEMENTATION POINTS**

**A. Counselling**

• Alcohol and drug counselling to be included in ART initiation and adherence counselling sessions. These sessions should also include information about interaction of ART, and alcohol and other substances

• Counselling module for disclosure of HIV status should be developed and implemented in TIs

• Family counselling modules should be developed (this may include biological families as well non-biological families). This module should discuss the role of families in ensuring approriate ART intake and management of HIV in KPs

• Group counselling sessions led by HIV infected KPs may be considered for repeat ART counselling. They can personalise the use of ART, follow-up, and address stigma and discrimination by other KPs as well as general community

**B. Administrative issues**

• Develop guidelines for lost ART bottles and implement it across all the ART centres

• Develop guidelines for incarcerated HIV infected individuals

• Change the package of ARTs so that ‘AIDS’ or ‘HIV’ are not inlcuded on the package in any form

• A one time cost of providing ‘pill reminder’ or ‘pill organiser’ may be considered. The total cost can be lowered by bulk purchase.

• Assign technically competent nurses/blood technicians for easier access of veins for blood investigations in PWIDs

• Guidelines to engage NGOs in providing ART to homeless KPs may be considered

• Digitise the records and reducing the use of green book and replace it by health cards. This may be useful to improve attendance, and reduce stigma and discrimination at health care centres

**C. Other suggestions**

• HIV prevention, care, and discrimination messages should be developed for digital platforms, online videos, and other media. These messages should be displayed throughout the year and not just around the World AIDS Day.

• Monitoring long term side effects of ART and association with different drug groups may be considered.
